# A Multi-center Cross-Sectional Assessment of Healthcare Professionals’ Knowledge, Attitudes, and Practices Toward Thromboprophylaxis

**DOI:** 10.7759/cureus.61835

**Published:** 2024-06-06

**Authors:** Feras Almarshad, Altaf Bandy, Abdurahman Alfaiz, Saad Fares Alotaibi, Shafi Ayidh Alaklabi, Yasser Faiz Alotaibi

**Affiliations:** 1 Internal Medicine, Shaqra University, Shaqra, SAU; 2 Community Medicine, Shaqra University, Shaqra, SAU; 3 College of Medicine, Shaqra University, Shaqra, SAU

**Keywords:** thromboprophylaxis, practice, knowledge, anticoagulants, ambulation walker

## Abstract

Background

Venous thromboembolism (VTE) is a potentially avoidable condition that affects hospitalized patients. Risk stratification and preventative strategies have substantial evidence supporting their use, but reasons hinder widespread adoption, compliance, and success, explaining the continuation of VTE. Thromboprophylaxis consists of several measures that are frequently adopted to avoid the complications of VTE. The study evaluated knowledge, attitude, and practice toward using thromboprophylaxis by health professionals.

Methods

This multi-center cross-sectional study was carried out on health professionals involved in patient care working in various secondary and tertiary hospitals in the study region between October 2023 and February 2024. A previously published questionnaire was sent in the form of an online survey to the study participants. Fifteen, ten, and nine questions evaluated the participants’ knowledge, attitude, and practice of thromboprophylaxis, respectively. The study followed the checklist for reporting results of the Internet E-survey (CHERRIES) guidelines. Frequency and percentages were calculated. Bi-variable and multi-variable logistic regression were carried out and presented as crude and adjusted odds ratios with corresponding 95% confidence intervals. A P-value of <0.05 was considered significant.

Results

Of the 219 participants, 115 (52.5%) and 104 (47.5%) were males and females. More than 50.7% were in the age group of >30 years, and the majority of the participants possessed a bachelor’s (104 (47.5%)) degree. One hundred seventy-six (80.4%) of the study participants were working in government hospitals, and the majority (112 (51.1%)) were nurses. One hundred sixty-two (74% (67.63-79.65)), 175 (79.9% (73.98-85.01)) and 211 (96.3% (92.93)) had satisfactory knowledge, a positive attitude, and good practice regarding thromboprophylaxis, respectively. Regarding the facility characteristics, 196 (89.5%), 150 (68.5%), and 164 (74.9%) respondents agreed with the availability of a VTE prevention policy, VTE prevention consultants, and the availability of anticoagulants. Eighty (36.5%) participants responded with a ‘not availability’ of pneumatic compression devices. Of the 15 knowledge questions, the majority (124 (56.6%)) participants faulted the false statements regarding ‘patients of DVT being symptomatic’ and 119 (54.3%) on the statement that helping patients ‘out of bed activity does not prevent VTE’. On multi-variable analysis, participants who were aware of having a VTE prevention policy and availability of anticoagulants were more knowledgeable with adjusted odds ratios of 5.39 (1.88-15.39) and 2.52 (1.12-5.63) respectively. Every practice domain received >90% approval ratings.

Conclusions

The study concludes that an overall satisfactory knowledge and positive attitude regarding thromboprophylaxis exists among the participants. The study proposes more training sessions on VTE prevention and orientation of health professionals on the availability of VTE policy guidelines and facility availability of resources for thromboprophylaxis.

## Introduction

Venous thromboembolism (VTE) is a critical medical condition that includes deep vein thrombosis (DVT) and pulmonary embolism (PE) [1]. In clinical practice, approximately two-thirds of VTE events present as DVT, while one-third as PE with or without DVT [2]. The incidence of VTE increases after hospitalization due to an increase in risk factors. Among hospitalized patients, 78% present with more than one risk factor for VTE, and about 20% have more than three risk factors [3]. The call to action aims to minimize hospital-acquired VTE by 20% by 2030 [4].

Thromboprophylaxis in VTE involves the use of various interventions, including pharmacological agents and mechanical methods, to reduce the risk of blood clot formation and subsequent complications [5]. This is especially important for individuals who are at a high risk of developing VTE, such as those undergoing surgery, bedridden patients, and individuals with certain medical conditions. Pharmacologic VTE prevention is related to a reduction in mortality [2]. Pharmacological agents act by inhibiting the clotting factors in the blood, thereby reducing the risk of clot formation. Anticoagulants, such as heparin or low molecular weight heparin, are commonly used for thromboprophylaxis. However, it is important to carefully consider the individual patient's risk factors and any contraindications before initiating pharmacological thromboprophylaxis [6]. By implementing thromboprophylaxis, healthcare providers can mitigate the potentially life-threatening consequences of VTE such as PE [7].

However, earlier studies have shown under-utilization of prophylactic thromboprophylaxis in eligible hospitalized patients. In these studies, the implementation of VTE thromboprophylaxis varied from 24% to 84% [8,9]. Further, there is a lack of consistency in the application of thromboprophylaxis among surgeons. Because of a lack of adequate VTE prevention, 10-80% of inpatients develop the condition, with mortality rates ranging from 30% to 40% [10]. The appropriate use of thromboprophylaxis in hospitalized patients is an important predictor of the risk of fatal PE. Health professionals play an important role in VTE prevention by determining the likelihood of VTE and prescribing necessary preventive measures. In addition, the effectiveness of thromboprophylaxis heavily relies on the knowledge and awareness of health professionals. In order to ensure the best possible outcomes for patients, it is essential that health professionals have a deep understanding of thromboprophylaxis guidelines, risk assessment tools, and appropriate prophylactic measures [6]. Further, staying updated with the latest research and recommendations in thromboprophylaxis is essential. This includes understanding the different types of thromboprophylaxis, such as pharmacological and mechanical measures, and being able to make informed decisions based on individual patient needs and risk factors [7]. It is also important for health professionals to recognize the signs and symptoms of potential thrombotic events in order to promptly initiate appropriate interventions [11]. Proper patient education regarding the importance of thromboprophylaxis and the signs of potential complications is vital for ensuring continuity of care beyond the hospital setting [12].

In the above context, a comprehensive understanding of thromboprophylaxis is crucial for health professionals to effectively mitigate the risk of thrombotic events in their patients. The lack of evidence on this topic in the study area prompted us to conduct the study with the objective of assessing knowledge attitudes and practices among healthcare providers regarding thromboprophylaxis across different hospitals in the third cluster hospitals in the Riyadh region of Saudi Arabia.

## Materials and methods

Study design, period, and setting

This multi-center cross-sectional study was conducted between October 2023 and February 2024 covering health professionals involved in patient care from secondary and tertiary hospitals of the third cluster hospitals in the Riyadh region of Saudi Arabia. Both male and female health professionals working in these hospitals were targeted in this study. All available doctors and nurses were included and other staff not directly involved in patient care were excluded from this study.

Sample size

The sample size was calculated by using an arbitrary fixed population of 400 health professionals working in these hospitals. Assuming that half of the study participants have good knowledge, attitude, and practice, we used the Raosoft sample size calculator to determine the sample size (http://www.raosoft.com/samplesize.html; Raosoft, Inc., Seattle, WA). At a confidence level of 95% with a 5% margin of error, 197 participants were required for this study. With a 10% non-response rate, an additional 20 participants were added to the sample size. The total sample size for this study was 217 individuals.

The study employed the consecutive sampling technique to reach out to the study participants. After receiving ethical approval from the Committee on Bioethics at Shaqra University, an online survey questionnaire was sent to all participants.

Study instrument

A pre-validated English questionnaire from a previous study was used [13]. There were 15 questions to assess the knowledge about thromboprophylaxis. The total knowledge score was 15 for correct answers. For a correct statement item if the participant answers ‘false’ or ‘I don’t know’, they were considered wrong answers and allotted zero and 1 for true and vice versa for incorrect statement items. The participant having >70% correct answers was labeled as having adequate knowledge. The attitude of health professionals was assessed by 10 questions on a five-point Likert scale. The score ranged from 15 to 50. A score of >70% was considered as positive attitude. Nine items addressed the practice of health professionals with a three-point Likert scale (always, sometimes, and never). Always and sometimes were considered as ‘practice’ and never as ‘no practice’. A total practice score of >70% was considered good practice.

Before using the questionnaire on the current population, it was pre-tested on 20 participants. The data of this pilot study was analyzed and a Cronbach’s alpha score of 0.85 for knowledge, 0.70 for attitude, and 0.75 for practice were observed. The questionnaire was deemed adequate for using in the current context. The data from this pilot study was excluded from the final analysis.

The questionnaire was prepared as a Google form. The first page of the questionnaire mentioned the purpose of the study, voluntariness of participation, confidentiality, anonymous data collection, number of questions to answer, and time required to complete the questionnaire followed by the consent statement. Participants who consented were able to proceed to complete the survey. The link to the questionnaire was emailed to study participants. The checklist for reporting results of internet E-Surveys (CHERRIES) was followed.

The questionnaire collected information on independent variables like gender, age, years of experience, education, working unit, etc. The dependent variables included knowledge (satisfactory or unsatisfactory), attitude (positive or negative), and practice (good or poor) regarding thromboprophylaxis.

Data analysis

The data was analyzed with Statistical Package for the Social Sciences (IBM SPSS Statistics for Windows, IBM Corp., Version 20.0, Armonk, NY). The categorical variables are presented as frequencies and percentages. Both bi-variable analysis and multi-variable logistic regression analysis were carried out. Binary logistic regression analysis was carried out for variables with a P-value <0.2. Crude and adjusted odds ratios were calculated with the corresponding 95% confidence intervals. A P-value of <0.05 was considered statistically significant.

## Results

Baseline characteristics of the study participants

A total of 219 participants responded to the e-questionnaire, comprising 115 (52.5%) males and 104 (47.5%) females. One hundred and eight (49.3%) participants were >30 years of age group. One hundred and four (47.5%; P<0.01) possessed a bachelor’s degree that was significantly higher compared to other qualifications. Similarly, nurses comprised significantly higher proportions (112 (51.1%); P<0.01). One hundred and seventy-six (80.4%; P<0.01) were working in government hospitals and most (127 (58.0%)) of the participants had an experience of less than five years (Table 1).

**Table 1:** Socio-demographic characteristics of the study population (n=219) * indicates statistical significance

Socio-Demographic Characteristics	Number (n)	Percent (%)	P-value
Gender			
Male	115	52.5	0.45
Female	104	47.5	
Age group (years)			
>30	108	49.3	0.83
≤30	111	50.7	
Educational level			
Bachelors	104	47.5	<0.01*
PG Diploma	43	19.6	
Masters	18	8.2	
PhD/Board/Fellowship	54	24.7	
Profession			
Nursing	112	51.1	<0.01*
General practitioners/residents	34	15.5	
Specialists	42	19.2	
Consultants	31	14.2	
Hospital			
Government	176	80.4	<0.01*
Private	43	19.6	
Working unit			
Internal medicine and allied	111	50.7	<0.01*
Intensive care unit	81	37.0	
Surgery and allied	27	12.3	
Experience (years)			
<5	127	58.0	<0.01*
5-10	42	19.2	
>10	50	22.8	

Overall knowledge of VTE prophylaxis

One hundred and sixty-two (74.0%; CI (67.62-79.65)) of the respondents had satisfactory knowledge, 175 (79.9%; CI (73.98-85.01)) had a positive attitude, and 211 (96.3%; CI (92.93-98.41)) were having good practice regarding thromboprophylaxis (Table 2).

**Table 2:** Overall knowledge, attitude, and practice toward thromboprophylaxis among health professionals (219)

Domain	Level	Number	Percent (95% CI)
Knowledge	Satisfactory	162	74.0 (67.63-79.65)
	Un-satisfactory	57	26.0 (20.35-32.37)
Attitude	Positive	175	79.9 (73.98-85.01)
	Negative	44	20.1 (14.99-26.02)
Practice	Good practice	211	96.3 (92.93-98.41)
	No practice	8	3.7 (1.59-7.07)

One hundred and ninety-six (89.5%) participants were aware of the availability of VTE guidelines followed by 150 (68.5%) about VTE-preventing consultants. The awareness of the availability of VTE anticoagulants was observed among 164 (74.9%), while only 132 (60.3%) respondents were aware of the availability of ambulation walkers (Table 3).

**Table 3:** Knowledge of facilities and guidelines among health professionals (n=219)

Variable	Available n (%)	Not Available n (%)
Venous Thromboembolism (VTE) Prevention Policy		
VTE preventing policy & guidelines	196 (89.5)	23 (10.5)
VTE preventing consultants	135 (61.6)	84 (38.4)
Training sessions for health professionals regarding VTE prevention	150 (68.5)	69 (31.5)
VTE Preventing Facilities and Supplies		
Ambulation walker	132 (60.3)	87 (39.7)
Intermittent pneumatic compression (IPC) device	139 (63.5)	80 (36.5)
Pharmacological measures preventing VTE (anticoagulant drugs)	164 (74.9)	55 (25.1)

Knowledge about venous thromboprophylaxis

Out of 15 knowledge questions, 13 statements were true, and two statements were false. The median knowledge score was 12.0; with a range of 2-15 (IQR 3.0). More than 80% of the participants answered correctly to 10 true statement questions. The two false statement questions that ‘out of bed activity’ does not prevent VTE and that most hospitalized patients ‘who develop DVT are symptomatic’ were answered incorrectly by 100 (45.7%) and 95 (43.4%) participants respectively (Table 4).

**Table 4:** Knowledge of participants about thromboprophylaxis (n=219) DVT: deep venous thrombosis; VTE: venous thromboprophylaxis; LMWH: low molecular weight heparin

Knowledge (15 Questions)	Correct Answers n (%)	Incorrect Answers n (%)
Knowledge score	12.0; Range: 2-15 (IQR 3.0)	
Instruct bedridden patients in lower limb exercises is helpful to prevent VTE (T)	197 (90.0)	22 (10.0)
Regularly moving bedridden patients is helpful for preventing VTE (T)	197 (90.0)	22 (10.0)
Adequate hydration can prevent the occurrence of VTE (T)	179 (81.7)	40 (18.3)
Controlling blood glucose and blood lipids helps to prevent VTE (T)	172 (78.5)	47 (21.5)
Helping hospitalized patients to perform out-of-bed activity as soon as their condition allows does not prevent VTE (F)	100 (45.7)	119 (54.3)
Elastic compression stockings may prevent DVT development (T)	178 (81.3)	41 (18.7)
Most hospitalized patients who develop DVT are symptomatic (F)	95 (43.4)	124 (56.6)
Patients with bed ridden/immobilized, prolonged surgery and previous DVT or PE should have DVT prophylaxis (T)	185 (84.5)	34 (15.5)
Patients with prophylactic doses of unfractionated heparin (5000 u BID) do not need to stop it before surgery or invasive procedures (T)	115 (52.5)	104 (47.5)
General anesthesia of more than 30 mins has a risk of DVT (T)	149 (68.0)	70 (32.0)
Obese patients and old age had more incidence of DVT during surgery (T)	195 (89.0)	24 (11.0)
Early ambulation after surgery may prevent DVT (T)	185 (84.5)	34 (15.5)
Intermittent pneumatic compression devices may prevent DVT development (T)	188 (85.8)	31 (14.2)
Selection of DVT preventive measures for surgical patients determined by the number of risk factors (T)	184 (84.0)	35 (16.0)
Heparin or LMWH may prevent DVT development (T)	181 (82.6)	38 (17.4)

Baseline characteristics that predict knowledge of thromboprophylaxis among the study participants

On bi-variable analysis, participants who were aware of the VTE prevention policy were eight times (CI 3.33-22.40) more knowledgeable compared to those who didn’t know. Similarly, participants who were aware of pharmacological measures for VTE prevention were three times (CI 1.99-7.42) more knowledgeable about thromboprophylaxis. On multi-variable analysis, these two variables (availability of VTE guidelines and policy aOR=5.39 (CI 1.88-15.39), presence of pharmacological measures aOR=3.84 (1.99-7.42)) continue to be significant factors predicting thromboprophylaxis knowledge among the participants (Table 5).

**Table 5:** Factors affecting thromboprophylaxis knowledge among health professionals (n=219) * indicates statistical significance

Socio-Demographic Characteristics	Total	Satisfactory Knowledge	Unsatisfactory Knowledge	Crude Odds Ratio (95% CI)	Adjusted Odds Ratio (95% CI)
Gender					
Male	115	85	30	0.99 (0.54-1.81)	0.94 (0.42-2.13)
Female	104	77	27		
Age in years					
>30	108	79	29	0.91 (0.50-1.68)	0.96 (0.41-2.24)
<30	111	83	28		
Education					
Bachelors	104	78	26	1	
PG diploma	43	33	10	1.1 (0.47-2.53)	1.03 (0.29-3.69)
Masters	18	14	4	1.16 (0.35-3.86)	0.45 (0.61-3.31)
PhD/Board/Fellowship	54	37	17	0.72 (0.35-1.49)	0.67 (0.16-2.80)
Profession					
Nursing	112	85	27	1	
General practitioners/residents	34	21	13	0.51 (0.22-1.16)	1.72 (0.40-7.39)
Specialists	42	32	10	1.01 (0.44-2.33)	1.23 (0.35-4.27)
Consultants	31	24	7	1.08 (0.42-2.80)	1.24 (0.26-5.84)
Hospital					
Government	176	131	45	1.13 (0.53-2.38)	0.54 (0.14-2.36)
Private	43	31	12		
Working unit					
Internal medicine and allied	111	80	31	1	
Intensive care unit	81	61	20	1.18 (0.61-2.27)	1.40 (0.63-3.12)
Surgery and allied	27	21	6	1.35 (0.50-3.67)	0.70 (0.23-2.12)
Experience in years					
<5	127	95	32	1	
5-10	42	34	8	1.43 (0.69-3.41)	1.30 (0.51-3.30)
>10	50	33	17	0.69 (0.32-1.32)	0.70 (0.23-2.12)
Venous Thromboembolism (VTE) Preventing Policy					
Available	196	155	41	8.64 (3.33-22.40)	5.39 (1.88-15.39)*
Not available	23	7	16		
Are pharmacological measures preventing VTE (anticoagulant drugs)?					
Present	164	133	31	3.84 (1.99-7.42)	2.52 (1.12-5.63)*
Absent	55	29	26		

Attitude toward thromboprophylaxis

Ten questions evaluated the attitude of health professionals on a 5-point Likert scale (1=strongly disagree to 5=strongly agree). The median attitude score was 39.0 (range 10-50; IQR 7.0). Seven attitude questions received an agreement of >75%. One hundred sixty-one (73.5%) of the participants believed in the effectiveness of currently used anticoagulant strategies. One hundred fifty-three (69.9%) and 106 (48.8%) believed that VTE prophylaxis is necessary before surgery and that the current anticoagulants are under or over-utilized respectively (Table 6).

**Table 6:** Attitude of the study participants toward thromboprophylaxis (n=219) DVT: deep venous thrombosis; VTE: venous thromboprophylaxis

Attitude Statements	Strongly Disagree n (%)	Disagree n (%)	Neutral n (%)	Agree n (%)	Strongly Agree n (%)
Attitude Score	39.0; Range 10-50 (IQR 7.0)				
I believe that using DVT preventive measures among hospitalized patients is beneficial	15 (6.8)	3 (1.4)	29 (13.2)	97 (44.3)	75 (34.2)
I believe that an assessment of DVT risk factors is necessary prior to surgery	12 (5.5)	12 (5.5)	21 (9.6)	106 (48.4)	68 (31.1)
I believe that the prevention/prophylaxis of DVT is necessary prior to surgery	17 (7.8)	8 (3.7)	41 (18.7)	89 (40.6)	64 (29.2)
I believe that educating patients regarding preventive measures of DVT is necessary	14 (6.4)	5 (2.3)	26 (11.9)	103 (47.0)	71 (32.4)
I believe that healthcare providers require training in methods to prevent DVT	13 (5.9)	7 (3.2)	22 (10.0)	97 (44.3)	80 (36.5)
I believe that currently used anticoagulant strategies for the prevention of VTE in hospitalized patients are effective	8 (3.7)	11 (5.0)	39 (17.8)	112 (51.1)	49 (22.4)
I believe that current anticoagulant strategies are under- or over-utilized	13 (5.9)	27 (12.3)	73 (33.3)	85 (38.8)	21 (9.6)
I believe that VTE risk must be assessed in hospitalized patients	12 (5.5)	5 (2.3)	22 (10.0)	109 (49.8)	71 (32.4)
I believe that VTE prophylaxis can improve the quality of medical care	13 (5.9)	3 (1.4)	22 (10.0)	113 (51.6)	68 (31.1)
I believe that a multi-disciplinary team must provide therapy to patients with VTE	11 (5.0)	8 (3.7)	26 (11.9)	115 (52.5)	59 (26.9)

Practice of thromboprophylaxis among the study participants

More than 90% of the study participants responded to practicing the various domains of practice statements. Almost 98.2% of the respondents were providing VTE information on risks and prevention, while 90.4% were administering anticoagulants as a preventive measure (Table 7).

**Table 7:** Practice of healthcare professionals toward thromboprophylaxis (n=219) DVT: deep venous thrombosis; VTE: venous thromboprophylaxis

Practice Statements	Always and Sometimes n (%)	Never n (%)
Practice Score	9.0; Range 0-9 (IQR 0.0)	
Providing information to patients or relatives about risks and prevention of DVT	215 (98.2)	4 (1.8)
Encouraging patients to do foot and leg exercises by themselves or relatives to help if patients are unable to do so	210 (95.9)	9 (4.1)
Encouraging early ambulation after surgery of patients	209 (95.4)	10 (4.6)
Assessing the DVT risks of patients regularly	212 (96.8)	7 (3.2)
Administering anticoagulants as preventive measure in clinic	198 (90.4)	21 (9.6)
Monitoring the side effects of the anticoagulants	213 (97.3)	6 (2.7)
Encouraging patients to elevate their legs	212 (96.8)	7 (3.2)
Educating the patients on sufficient fluid intake	211 (96.3)	8 (3.7)
Assessing the patients regularly for signs and symptoms of DVT/VTE	212 (96.8)	7 (3.2)

## Discussion

Hospitalization contributes up to 60% of all VTE cases, either during admission or within 90 days following discharge [10,14]. However, VTE is preventable and morbidity and mortality linked with hospitalization can be avoided [15]. Healthcare professionals play a major role in the prevention of VTE. Understanding the impact of VTE on patient outcomes goes beyond its direct association with mortality and complications. It encompasses a broader spectrum of consequences, including prolonged hospital stays, decreased quality of life, and the financial burden on patients and healthcare systems [2].

In the current study, the overall satisfactory knowledge was observed in 74.0% (CI (67.62-79.65)) of the respondents. An earlier study from Saudi Arabia has reported a satisfactory knowledge score of 86.8% among surgeons which is in agreement with our findings [16]. However, our findings are better than another study that has reported a satisfactory knowledge of 49.8% [13]. The higher knowledge scores in our study can be attributed to the secondary and tertiary healthcare setting from which the study population was drawn. The other reason could be that our study comprised 52.1% of nurses and 37.0% of our sample were working in ICUs compared to 62.4% of nurses and 4.5% working in ICUs in the earlier study. Our results on knowledge scores are also higher compared to a study conducted on the medical staff in China that reported a knowledge score of 57.6% [17]. This variation could stem from differences in the tools used for evaluating knowledge, the composition of the study population, and differences in the professional and educational levels.

On multi-logistic regression, the study found an association between various baseline demographic characteristics like higher age group, education level, professional position (general physicians versus consultants), employed in the government sector and working in ICU and surgical wards, and satisfactory knowledge but did not reach the statistical significance. The study found that the participants having information about the availability of VTE policy (OR=8.64 (3.33-22.40); aOR=5.39 (1.88-15.39)) and presence of anticoagulants (OR=3.84 (1.99-7.42); aOR=2.52 (1.12-5.63)) were more likely to have a significant association with satisfactory knowledge about VTE. Our findings are in line with an earlier study from Northern Cyprus that reported, nurses >31 years of age, >11 years of experience, and working in ICUs had significantly higher median scores of knowledge [18]. A study from China reported that the nurses from the ICU department obtained the highest knowledge scores, while those from the pediatrics department scored the lowest. Higher education levels, professional ranks, and nursing experience were significantly associated with higher knowledge scores [19].

The study observed an overall attitude score of 79.9 (73.98-85.01) with a median attitude score was 39.0 (range: 10-50; IQR 7.0). Our results are in line with two studies mentioned subsequently. An earlier study has reported 75% [20] of acceptable attitude levels among orthopedic surgeons while another study report from China observed a median affirmative rate of 99.0% for attitudinal items [17]. An earlier study from Saudi Arabia has reported a positive attitudinal score of 84.27% of surgeons [16].

Our study observed that 96.3% (92.93-98.41) of the study participants had good practice toward VTE prophylaxis. This frequency of good practice is higher than the one reported in a study from China, where it has been reported to be just 52.3% among ICU staff for mechanical thromboprophylaxis [21]. In a study reported from Al-Najaf, it was reported that only 63.2% of the obstetricians and gynecologists claimed to follow specific thromboprophylaxis guidelines [22]. In a similar study, 85% of the surgeons responded that they prescribe thromboprophylaxis. The prescription rate varied among different settings; highest in ICU (81%) patients while 54.4% among post-operative settings [20]. Differences in thromboprophylaxis practices among different populations may be due to a couple of reasons, which include differences in the questionnaire used to assess knowledge and attitude, variability in practicing guidelines, diversity of patients and their risk factors, differences in clinical presentations of the patients, and so on. Furthermore, these variations could also be viewed in the context of a lack of uniformity in the curriculum taught at the graduate and undergraduate levels, differences in the established clinical practices, and different training guidelines.

Limitations

The generalizability of findings is constrained by the cross-sectional design of the study which limits causal relationship. Further, generalizability is limited by the geographical location, setting, and composition of the study sample which varies between studies. The participants in the current study were from secondary and tertiary hospitals and the results can be extrapolated to healthcare professionals from these institutions only. In addition, reliance on self-reported data may introduce response bias.

## Conclusions

The majority of respondents displayed satisfactory knowledge about thromboprophylaxis. Their attitude toward thromboprophylaxis is predominantly positive, which aligns with their practices. The high rates of good practice in informing patients about risks and administering anticoagulants reflect a commitment to evidence-based care and patient education as integral components of thromboprophylaxis.
